# Supplementary material for: The role of Rif1 in telomere length regulation is separable from its role in origin firing
Source: eLife. 2020 Jun 29;9:e58066. doi: 10.7554/eLife.58066 (PMC7371424; doi:10.7554/eLife.58066)
Supplement: Source code 1. [file elife-58066-code1.zip › Sourcecode1.pdf]

qsub runConcatenate.sh

Data was first uploaded in folders with forward and reverse reads, and then concatenated Example below:

```
#!/bin/sh
#$ -N move
#$ -S /bin/sh
#$ -cwd
#$ -q zappa
#$ -l mem_free=2G,h_vmem=3G

mkdir Seq3_151_alpha; cat Seq3_151_alpha*_R1_* > Seq3_151_alpha/Seq3_151_alpha_R1.fastq.gz; cat Seq3_151_alpha*_R2_* > Seq3_151_alpha/Seq3_151_alpha_R2.fastq.gz
mkdir Seq3_151_HU; cat Seq3_151_HU*_R1_* > Seq3_151_HU/Seq3_151_HU_R1.fastq.gz; cat Seq3_151_HU*_R2_* > Seq3_151_HU/Seq3_151_HU_R2.fastq.gz
```

run_build_bwa_index.sh

Indexed sacCer3 to align reads

```
#!/bin/sh
#$ -N bwaindex
#$ -r n
#$ -j yes
#$ -S /bin/sh
#$ -cwd
#$ -q zappa
#$ -l mem_free=6G,h_vmem=8G

module load sharedapps bwa

wget http://hgdownload.cse.ucsc.edu/goldenPath/sacCer3/bigZips/chromFa.tar.gz
tar xvzf chromFa.tar.gz
cat chr*fa > sacCer3.fa
bwa index sacCer3.fa
mkdir Chromosomes
mv chr* Chromosomes/
```

samplenames.txt

Example below:

```
009_Seq2_191_alpha
010_Seq2_191_HU
```

runBWA.sh

```
#!/bin/sh
#$ -N BWA-MEM
#$ -S /bin/sh
#$ -cwd
#$ -q zappa
#$ -l mem_free=12G,h_vmem=15G
#$ -pe zappa-pe 3,6

. /etc/profile.d/modules.sh

module load sharedapps sge bwa samtools sambamba bamtools

mypath=`echo /path/${projectid}_${in}`
samplename=`echo $in | sed -e s/^..._//`

echo $TMPDIR
cd $TMPDIR

cp $mypath/${samplename}_R1.fastq.gz .
cp $mypath/${samplename}_R2.fastq.gz .

ls -l

bwa mem -M -t $NSLOTS /path/sacCer3.fa ${samplename}_R1.fastq.gz ${samplename}_R2.fas
tq.gz | samtools view -bS - > ${samplename}_bwa_sacCer3.bam
sambamba sort -t $NSLOTS ${samplename}_bwa_sacCer3.bam -o ${samplename}_bwa_sacCer3_s
orted.bam
samtools index ${samplename}_bwa_sacCer3_sorted.bam
samtools flagstat ${samplename}_bwa_sacCer3_sorted.bam > ${samplename}_bwa_sacCer3.st
ats
cp ${samplename}_bwa_sacCer3_sorted.bam* /path/${projectid}_000_analysis/alignments/
cp ${samplename}_bwa_sacCer3.stats /path/${projectid}_000_analysis/stats/
```

runMarkDuplicates.sh

```
#!/bin/sh
#$ -N markDuplicates
#$ -S /bin/sh
#$ -cwd
#$ -q zappa
#$ -l mem_free=12G,h_vmem=15G

. /etc/profile.d/modules.sh

module load sharedapps java samtools

samplename=`echo $in | sed -e s/^..._//`

java -Xmx8G -jar /path/picard-tools-1.119/MarkDuplicates.jar VALIDATION_STRINGENCY=LE
NIENT REMOVE_DUPLICATES=true ASSUME_SORTED=true CREATE_INDEX=true I=${samplename}_bwa
_sacCer3_sorted.bam O=${samplename}_bwa_sacCer3_DeDuplicated_sorted.bam M=../stats/${
samplename}_bwa_sacCer3_DeDuplicated_metrics.txt
samtools depth ${samplename}_bwa_sacCer3_DeDuplicated_sorted.bam | awk '{sum+=$3} END
{ print "Average = ",sum/NR}' > ../stats/${samplename}_sacCer3_avg_cov.txt
mv ${samplename}_bwa_sacCer3_DeDuplicated_sorted.bai ${samplename}_bwa_sacCer3_DeDupl
icated_sorted.bam.bai
```

runCreateReferenceBed.sh

windowsize=250

```
#!/bin/sh
#$ -N refBed
#$ -r n
#$ -j yes
#$ -S /bin/sh
#$ -cwd
#$ -q zappa
#$ -l mem_free=6G,h_vmem=8G

/path/createBED.pl $windowsize | sort -k1,1 -k2,2n > /path/sacCer3_${windowsize}.bed
```

createBED.pl

```
#!/usr/bin/perl

if ((@ARGV-0) < 1)
{
die ("provide window size\n");
}

%chrSize =();

open (IN, "/path/sacCer3.chrom.sizes");
while ($line= <IN>) {
chomp($line);
@data = split (/\t/, $line);
$chrSize{$data[0]} = $data[1];
}

while (($key, $value) = each(%chrSize)){
$start = 1;
$end = $ARGV[0];
while ($end < $value) {
print "$key\t$start\t$end\n";
$start += $ARGV[0];
$end += $ARGV[0];
}
print "$key\t$start\t$value\n";
}
```

runGetCount.sh

```
#!/bin/sh
#$ -N count
#$ -S /bin/sh
#$ -cwd
#$ -q zappa
#$ -l mem_free=2G,h_vmem=3G

. /etc/profile.d/modules.sh

for i in ../*/*_R1.fastq.gz
do
R1count=`zcat $i | wc -l`; expr $R1count / 4 >> temp.R1_readCount.txt
done

for i in ../*/*_R2.fastq.gz
do
R2count=`zcat $i | wc -l`; expr $R2count / 4 >> temp.R2_readCount.txt
done

paste samplenames.txt temp.R1_readCount.txt temp.R2_readCount.txt > ../Read_count.txt
rm temp*
```

bedGraphList.txt

Example below, used with the next script HU:alpha values from the Read_count.txt file created with the runGetCount.sh script

```
Seq2_191:181564466:179205324:250:Seq2_191_HU_vs_alpha_250_coverage.bedGraph
```

runCreateBedGraph.sh

```
#!/bin/bash
#$ -N CreateBedGraph
#$ -j yes
#$ -S /bin/sh
#$ -cwd
#$ -q zappa
#$ -l mem_free=4G,h_vmem=5G

. /etc/profile.d/modules.sh

module load sharedapps bedtools

patientname=`echo ${in} | cut -d: -f1`
readcount1=`echo ${in} | cut -d: -f2`
readcount2=`echo ${in} | cut -d: -f3`
windowsize=`echo ${in} | cut -d: -f4`
bedGraphFilename=`echo ${in} | cut -d: -f5`

bedtools multicov -bams /path/${patientname}_HU_bwa_sacCer3_DeDuplicated_sorted.bam -bed /path/sacCer3_${windowsize}.bed > ${patientname}_HU_${windowsize}_coverage.bed
bedtools multicov -bams /path/${patientname}_alpha_bwa_sacCer3_DeDuplicated_sorted.bam -bed /path/sacCer3_${windowsize}.bed > ${patientname}_alpha_${windowsize}_coverage.bed
/path/createBedGraphRatio.pl ${patientname}_HU_${windowsize}_coverage.bed ${patientname}_alpha_${windowsize}_coverage.bed $readcount1 $readcount2 $windowsize $bedGraphFilename
```

createBedGraphRatio.pl

```
#!/usr/bin/perl

if ((@ARGV-0) < 6)
{
   die ("provide sample1name, sample2name, sample1 read-count, sample2 read-count, wi
ndowsize & output filename\n");
}

open (IN1, "$ARGV[0]") || die ("The $ARGV[0] file doesn't exit\n");
open (IN2, "$ARGV[1]") || die ("The $ARGV[1] file doesn't exit\n");
open (OUT, ">$ARGV[5]");
print OUT "track type=bedGraph\n";
while (!eof IN1 and !eof IN2) {
    $line1 = <IN1>;
    chomp($line1);
    @data1 = split (/\t/, $line1);
    $line2 = <IN2>;
    chomp($line2);
    @data2 = split (/\t/, $line2);
    $coverage1 = $data1[3]/$ARGV[2];
    $coverage2 = $data2[3]/$ARGV[3];
    $coverageRatio = sprintf("%.2f", $coverage1/($coverage2+0.000000001));
    print OUT "$data1[0]\t$data1[1]\t$data1[2]\t$coverageRatio\n";
    }
```
