# Supplementary material for: The role of Rif1 in telomere length regulation is separable from its role in origin firing
Source: eLife. 2020 Jun 29;9:e58066. doi: 10.7554/eLife.58066 (PMC7371424; doi:10.7554/eLife.58066)
Supplement: Transparent reporting form [file elife-58066-transrepform.docx]

***eLife’s* transparent reporting form**

We encourage authors to provide detailed information *within their submission* to facilitate the interpretation and replication of experiments. Authors can upload supporting documentation to indicate the use of appropriate reporting guidelines for health-related research (see [EQUATOR Network](http://www.equator-network.org/%20)), life science research (see the [BioSharing Information Resource](https://biosharing.org/)), or the [ARRIVE guidelines](http://www.plosbiology.org/article/info:doi/10.1371/journal.pbio.1000412) for reporting work involving animal research. Where applicable, authors should refer to any relevant reporting standards documents in this form.

If you have any questions, please consult our Journal Policies and/or contact us: [editorial@elifesciences.org](mailto:editorial@elifesciences.org).

**Sample-size estimation**

- You should state whether an appropriate sample size was computed when the study was being designed
- You should state the statistical method of sample size computation and any required assumptions
- If no explicit power analysis was used, you should describe how you decided what sample (replicate) size (number) to use

We did not estimate a sample size or do a power calculation because all of our statistics were done on a fixed number of established replication origins in yeast. We analyzed 399 of the established replication origins that are reported in OriDB.

Please outline where this information can be found within the submission (e.g., sections or figure legends), or explain why this information doesn’t apply to your submission:

**Replicates**

- You should report how often each experiment was performed
- You should include a definition of biological versus technical replication
- The data obtained should be provided and sufficient information should be provided to indicate the number of independent biological and/or technical replicates
- If you encountered any outliers, you should describe how these were handled
- Criteria for exclusion/inclusion of data should be clearly stated
- High-throughput sequence data should be uploaded before submission, with a private link for reviewers provided (these are available from both GEO and ArrayExpress)

Please outline where this information can be found within the submission (e.g., sections or figure legends), or explain why this information doesn’t apply to your submission:

- Mcm4 phospho-shift western blots all had at least two technical replicates, for some genotypes more than two.
- Protein stability westerns were done on 2 biological replicates, and at least two technical replicates, often more, were carried out.
- Telomere length Southerns all were done on 2 biological replicates, and at least two technical replicates, often more, were carried out.
- Whole genome sequencing experiments were performed two biological replicates for most genotypes. For some a single biological sample was performed (*rif1-∆1322*, *rif1_HOOK_,* ∆N-dbf4 Cdc7 o/e).

**Statistical reporting**

- Statistical analysis methods should be described and justified
- Raw data should be presented in figures whenever informative to do so (typically when N per group is less than 10)
- For each experiment, you should identify the statistical tests used, exact values of N, definitions of center, methods of multiple test correction, and dispersion and precision measures (e.g., mean, median, SD, SEM, confidence intervals; and, for the major substantive results, a measure of effect size (e.g., Pearson's r, Cohen's d)
- Report exact p-values wherever possible alongside the summary statistics and 95% confidence intervals. These should be reported for all key questions and not only when the p-value is less than 0.05.

Please outline where this information can be found within the submission (e.g., sections or figure legends), or explain why this information doesn’t apply to your submission:

We describe the statistical tests used for quantifying relative origin firing scores in the methods section. The figure legends report the P value.

- Biologically we are interested in whether origin firing is increased in our mutants compared to WT
- Relative origin firing scores were displayed as dot plots and significance was analyzed using a one-sided non-parametric Wilcoxon signed rank test
- We analyzed 399 of the established replication origins that are reported in OriDB
- The origin firing data was not assumed to be a normal distribution
- The null hypothesis was that the median=0
- The alternative hypothesis was that the median is greater than 0

(For large datasets, or papers with a very large number of statistical tests, you may upload a single table file with tests, Ns, etc., with reference to sections in the manuscript.)

**Group allocation**

- Indicate how samples were allocated into experimental groups (in the case of clinical studies, please specify allocation to treatment method); if randomization was used, please also state if restricted randomization was applied
- Indicate if masking was used during group allocation, data collection and/or data analysis

Please outline where this information can be found within the submission (e.g., sections or figure legends), or explain why this information doesn’t apply to your submission:

N/A

**Additional data files (“source data”)**

- We encourage you to upload relevant additional data files, such as numerical data that are represented as a graph in a figure, or as a summary table
- Where provided, these should be in the most useful format, and they can be uploaded as “Source data” files linked to a main figure or table
- Include model definition files including the full list of parameters used
- Include code used for data analysis (e.g., R, MatLab)
- Avoid stating that data files are “available upon request”

Please indicate the figures or tables for which source data files have been provided:

We have provided all of the data used to generate each of the graphs in every figure in supplemental tables 2 and 3.
